# Paeoniflorin suppresses the apoptosis and inflammation of human coronary artery endothelial cells induced by oxidized low-density lipoprotein by regulating the Wnt/β-catenin pathway

**DOI:** 10.1080/13880209.2023.2220360

**Published:** 2023-09-06

**Authors:** Shasha Liu, Ying Li, Caojie Wu

**Affiliations:** Department of Geriatrics, Sichuan People’s Hospital, Sichuan Academy of Medical Sciences, Chengdu, Sichuan, China

**Keywords:** Coronary artery disease, proliferation, plaque lesion, blood lipid, interleukin

## Abstract

**Context:**

Paeoniflorin (PF) contributes to improving coronary artery disease (CAD).

**Objective:**

This study clarified the efficiency of PF in CAD and the molecular mechanism.

**Materials and methods:**

Human coronary artery endothelial cells (HCAECs) were treated with oxidized low-density lipoprotein (ox-LDL; 20, 40, 80 and 160 μg/mL) and PF (0.05, 0.1 0.2 and 0.4 mM). To study cell phenotypes, HCAECs were treated with 80 μg/mL ox-LDL with or without 0.1 mM PF for 24 h, and cell viability and apoptosis were evaluated using the methyl thiazolyl tetrazolium (MTT) assay and flow cytometry, respectively. In addition, inflammatory cytokines levels were measured by enzyme-linked immunosorbent assay (ELISA). Western blot evaluated the Wnt/β-catenin pathway-related factors.

**Results:**

ox-LDL and PF (0.2 and 0.4 mM) suppressed cell viability in a dose-dependent manner. The IC_50_ value of PF was 722.9 nM. PF facilitated cell viability (115.76%), inhibited apoptosis (46.28%), reduced IL-6 (63.43%) and IL-8 (66.70%) levels and increased IL-10 levels (181.15%) of ox-LDL-treated HCAECs. Additionally, PF inactivated the Wnt/β-catenin pathway, and XAV939 treatment further promoted cell viability (120.54%), suppressed apoptosis (56.92%), reduced the levels of IL-6 (76.16%) and IL-8 (86.82%) and increased the IL-10 levels (120.22%) of ox-LDL-induced HCAECs after PF treatment. Moreover, PF alleviated plaque lesions of the aorta and aorta root and serum lipid of ApoE^−/−^ mice with a high-fat diet.

**Discussion and conclusions:**

This study first revealed that PF inhibited ox-LDL-induced HCAECs apoptosis and inflammation *via* the Wnt/β-catenin pathway and alleviated CAD, suggesting the potential of PF as a drug for CAD treatment.

## Introduction

Cardiovascular disease is still a leading cause of death globally, featuring increasing and younger morbidity in developing countries (Fioranelli et al. 2018; Malakar et al. 2019). Coronary artery disease (CAD) refers to the situation with atherosclerosis in the coronary arteries. Abnormal lipid metabolism leads to lipid deposition and forms porridge-like white spots, inducing atherosclerosis (Efe et al. 2015). As the plaque increases, the blood circulation is blocked, thereby resulting in myocardial ischaemia and hypoxia and inducing CAD (Wang et al. 2018). Furthermore, CAD is the risk factor for heart failure and coronary embolism (Lala and Desai 2014; Narula et al. 2020), but the pathogenesis of which has not been fully elucidated.

Endothelial cells (ECs) are parts of the heart and vascular system involved in promoting new blood vessel formation and maintaining vascular tone (Bhasin et al. 2010). Damage, overactivation and dysfunction of ECs are the pathogenesis of the occurrence and progression of CAD (Sturtzel 2017). Abnormal proliferation and apoptosis of ECs are the main cause of endothelial dysfunction (Song et al. 2016). In this case, maintaining the normal proliferation and preventing apoptosis of ECs can alleviate endothelial dysfunction and decelerate the progression of CAD.

Paeoniflorin (PF), a pinane monoterpene glycoside, exists in all plants of the Paeoniaceae family and can be found in all parts of the plant with the highest content in roots (Xiang et al. 2020). PF has been reported to have a variety of biological activities, including anticancer, anti-inflammatory, hypolipidemic and antithrombotic effects (Yang et al. 2004; Xie et al. 2017; Xin et al. 2019; Xiang et al. 2020). Accumulating evidence has revealed that PF contributes to improving cardiovascular diseases, such as ischaemic injury, hypertension and stroke (Han et al. 2016; Yu et al. 2020; Tang H et al. 2021), and can attenuate atherosclerosis by reducing the production of cytokines, inhibiting apoptosis and promoting the proliferation of vascular smooth muscle cells (VSMCs; Guo et al. 2017; Li et al. 2017). However, the effects of PF on the proliferation and apoptosis of human coronary artery endothelial cells (HCAECs) remain unclear.

The components of the Wnt/β-catenin pathway are commonly mutated in numerous diseases that regulate cell differentiation, proliferation and apoptosis. The Wnt family contains 19 glycoproteins that are highly conserved in multiple specimens. Wnt ligands could bind to the complex of Frizzled/LRP5/6, leading to the activation of the cytosolic β-catenin (Zhou et al. 2014). The classical Wnt/β-catenin pathway activates transcription of downstream genes such as Cyclin D1 and c-myc by promoting β-catenin nuclear translocation (Zhao et al. 2020). This pathway is involved in the progression of CAD and related to the dysfunction of ECs (Liu et al. 2020, 2021). Thus, it is interesting to study whether PF regulates cell phenotype through this pathway.

Herein, the role of PF in the progression of CAD was explored, and the results showed that PF facilitated the proliferation and suppressed apoptosis and inflammation of oxidized low-density lipoprotein (ox-LDL)-induced HCAECs. The findings provide insight into CAD therapy.

## Materials and methods

### Cell culture

HCAECs (ScienCell, Carlsbad) were cultured in the Endothelial Cell medium (ECM; ScienCell) at 37 °C with 5% CO_2_.

**Figure 1.**
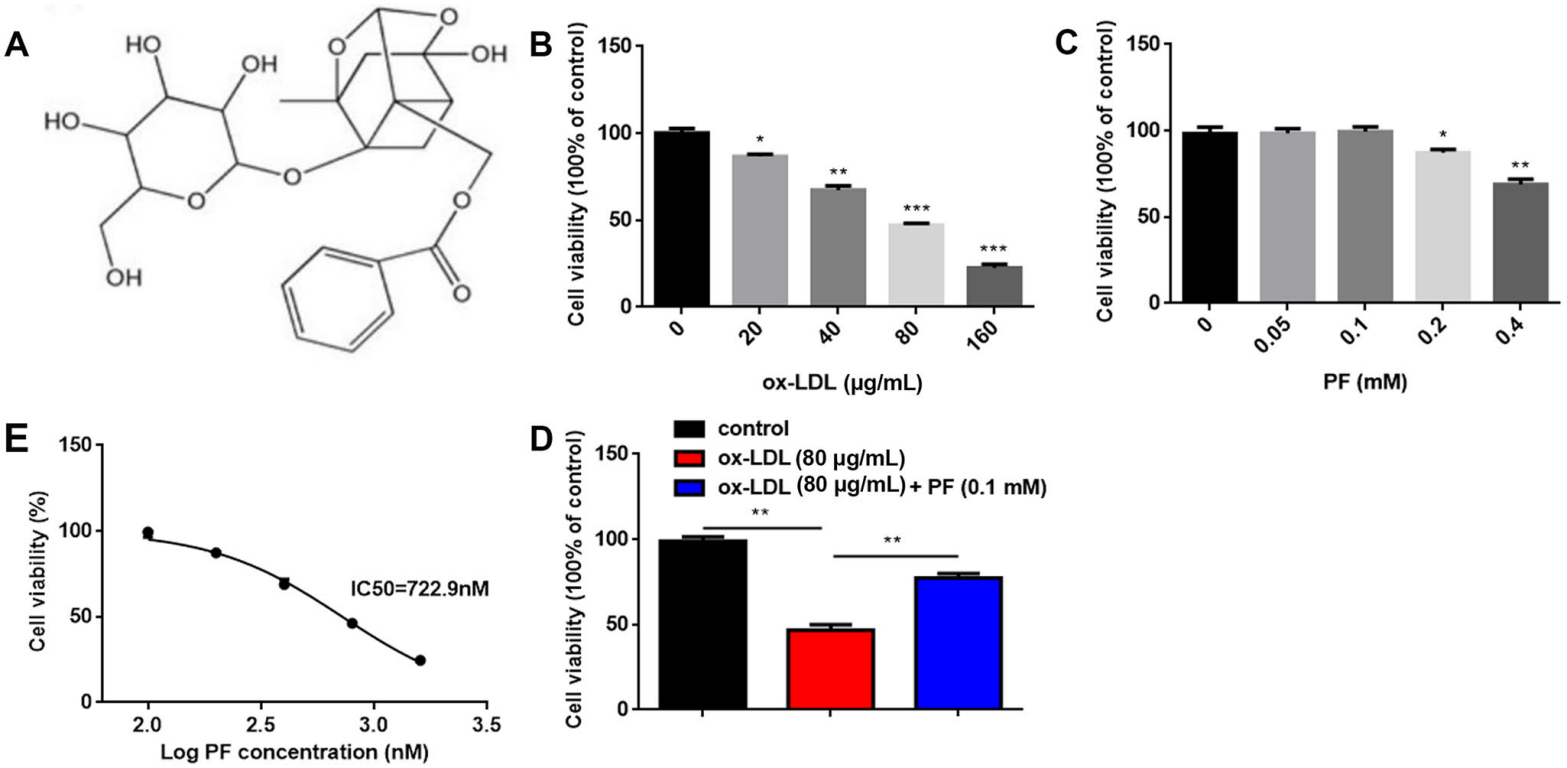
Effects of ox-LDL and PF on cell viability. (A) Chemical structure of PF; (B) the HCAECs were treated with 0, 20, 40, 80 and 160 μg/mL ox-LDL, and cell viability was examined using the MTT assay; (C) the HCAECs were treated with 0, 0.05, 0.1, 0.2 and 0.4 mM PF, and cell viability was assessed by the MTT assay; (D) the IC_50_ value of PF and (E) cell viability was evaluated by the MTT assay after treatment with 80 μg/mL ox-LDL and 0.1 mM PF. *p < 0.05, **p < 0.01 and ***p < 0.001.

To establish an injury model, the HCAECs were exposed to different concentrations of ox-LDL (0, 20, 40, 80 and 160 µg/mL; Sigma-Aldrich, St. Louis, MO, USA) for 24 h. PF (purity ≥98%; Figure 1(A)) was acquired from Sigma-Aldrich. The cells were treated with various concentrations of PF (0, 0.05, 0.1, 0.2 and 0.4 mM) for 24 h. Besides, the XAV939 (the Wnt/β-catenin pathway inhibitor; purity ≥98%) was purchased from Abcam (Cambridge), and the HCAECs were treated with 5 µM XAV939 for 6 h.

### Methyl thiazolyl tetrazolium (MTT) assay

The HCAECs (2 × 10^3^ cells/well) were seeded into 96-well plates and cultured for 0, 24, 48 and 72 h at 37 °C to assess cell proliferation. Then, 10 μg MTT solution (Sigma‐Aldrich) was incubated with the cells for 4 h. After that, 150 μL dimethylsulphoxide (DMSO) was incubated with the cells for 10 min, and the optical density (OD) value was tested at 490 nm.

### Flow cytometry

The Annexin‐V‐PE apoptosis kit (Invitrogen, Carlsbad) was used to analyse cell apoptosis. Briefly, the HCAECs were suspended in tubes using the Annexin-V binding buffer after being washed with PBS. Annexin-V PE (5 μL) and 7-AAD buffer (10 μL) were added to each tube to incubate with the cells for 15 min in the dark. After 400 μL Annexin-V binding buffer was added, apoptosis was analysed using flow cytometry.

### Enzyme-linked immunosorbent assay (ELISA) analysis

After the cells were treated with ox-LDL and PF, the release of inflammatory cytokines [interleukin (IL)-6, IL-8 and IL-10] was assessed using the specific ELISA kits (Abcam) following the protocol provided by the manufacturer.

### Western blot

The HCAECs were washed with PBS, and radioimmunoassay (RIPA) lysis buffer (Pierce, Rockford) was used to lyse the cells. The concentration of isolated proteins was detected using the BCA Protein Assay Kit (Pierce). Then, the proteins were subjected to 10% sodium dodecyl sulphate-polyacrylamide gel electrophoresis (SDS-PAGE) and transferred to the polyvinylidene fluoride (PVDF) membranes (Bio-Rad, Hercules). After being blocked with 5% skim milk for 1 h, the membranes were incubated with primary antibodies (anti-Bax: ab182733 1/2000; anti-Bcl2: ab182858 1/2000, anti-GAPDH: ab9485 1/2500, anti-β-catenin: ab32572 1/5000, anti-c-myc: ab32072 1/1000, anti-cyclin D1: ab16663 1/200, anti-E-cadherin: ab40772 1/10,000, Abcam) at 4 °C overnight and then incubated with secondary antibody (HRP conjugated goat anti-rabbit IgG: ab205718 1/5000, Abcam) at room temperature for 1 h. Protein bands were visualized using the ECL reagent (Pierce).

#### Animal model establishment

The animal study was approved by the Ethics Committee of Sichuan People’s Hospital (Approval number: 2018-310). All operations were performed following the *Guide* for the care and use of laboratory animals (National Research Council et al. 2010).

ApoE^−/−^ mice on a C57BL/6 background (male, six-week-old, Charles River, Beijing) were randomly divided into three groups, i.e., the control group, the high fat group and the high fat + PF group, with six mice per group. Mice in the high-fat group were fed with a high-fat diet for 12 weeks. The mice in the high fat + PF group were fed with a high-fat diet composed of conventional feed, 21% fat (wt/wt) and 0.15% cholesterol (wt/wt) (Beijing Keao Xieli Feed Co., LTD. Beijing) for 12 weeks and treated with 50 mg/kg PF intragastric administration twice a week. All mice were housed in specific pathogen-free (SPF) conditions with 12 h light/dark and 20–25 °C temperature. The body weight of mice was measured every two weeks. The venous blood was obtained from all mice on the last day of model establishment, and the mice were then perfused with PBS under anaesthesia to obtain the whole aorta and heart.

#### Determination of serum lipid

Serum was separated from the blood after centrifugation at 4000 rpm for 10 min.

According to the instructions from the manufacturer, cholesterol, low-density lipoprotein (LDL) cholesterol, high-density lipoprotein (HDL) cholesterol and triglycerides were measured using the total cholesterol assay kit, LDL cholesterol kit, HDL cholesterol kit and triglycerides assay kit (JianCheng, Nanjing), respectively.

#### Oil red O staining assay

The whole aorta was washed with PBS and fixed with 4% paraformaldehyde for 12 h. After longitudinal dissection, the aorta was soaked in 70% ethanol for 2 min and subsequently stained with Oil red O solution for 30 min. The results were visualized under a microscope. The plaque area was quantified using the Image-Pro Plus software.

The heart was fixed with 4% paraformaldehyde for 48 h, and the aortic root was obtained, which was serially sectioned to be frozen, followed by Oil red O staining as mentioned above. The lesion area was also quantified using the Image-Pro Plus software.

#### Immunofluorescence (IF) assay

Macrophage infiltration was analysed by detecting macrophage markers F4/80 using the IF assay. The paraffin sections of aortic root were dewaxed and rehydrated. After blocking using PBST with 1% BSA at room temperature for 30 min, the sections were incubated with anti-F4/80 (ab6640, 1:100, Abcam) overnight at 4 °C and incubated with Goat Anti-Rat IgG H&L (Alexa Fluor® 488) (ab150165, 1:1000, Abcam) for 1 h at room temperature. The nuclei were stained using DAPI. The results were observed under a fluorescence microscope (Olympus, Japan).

### Statistical analysis

Data from three independent experiments were analysed using GraphPad Prism 7.0 software and expressed as mean ± standard deviation (SD). Comparisons between the two groups were analysed using the Student’s *t*-test. Comparisons in multiple groups were analysed by one-way analysis of variance (ANOVA), with *p* < 0.05 indicating a significant difference.

## Results

### Effects of ox-LDL and PF on cell viability

The HCAECs were exposed to different concentrations of ox-LDL (0, 20, 40, 80 and 160 μg/mL). The results of the MTT assay showed that ox-LDL significantly inhibited cell viability in a dose-dependent manner (Figure 1(B)). Besides, the injury model of HCAECs was established using Ox-LDL at the density of 80 μg/mL and treated with several concentrations of PF (0, 0.05, 0.1, 0.2 and 0.4 mM). Cell viability was not affected by PF at 0, 0.05 and 0.1 mM, but significantly suppressed by PF at 0.2 and 0.4 mM (Figure 1(C)). The IC_50_ value of PF was 722.9 nM (Figure 1(D)). Thus, 0.1 mM PF was used in the following studies. Moreover, PF reversed the inhibition of cell viability induced by ox-LDL (Figure 1(E)).

### PF promotes cell viability, inhibits apoptosis and inflammation induced by ox-LDL

**Figure 2.**
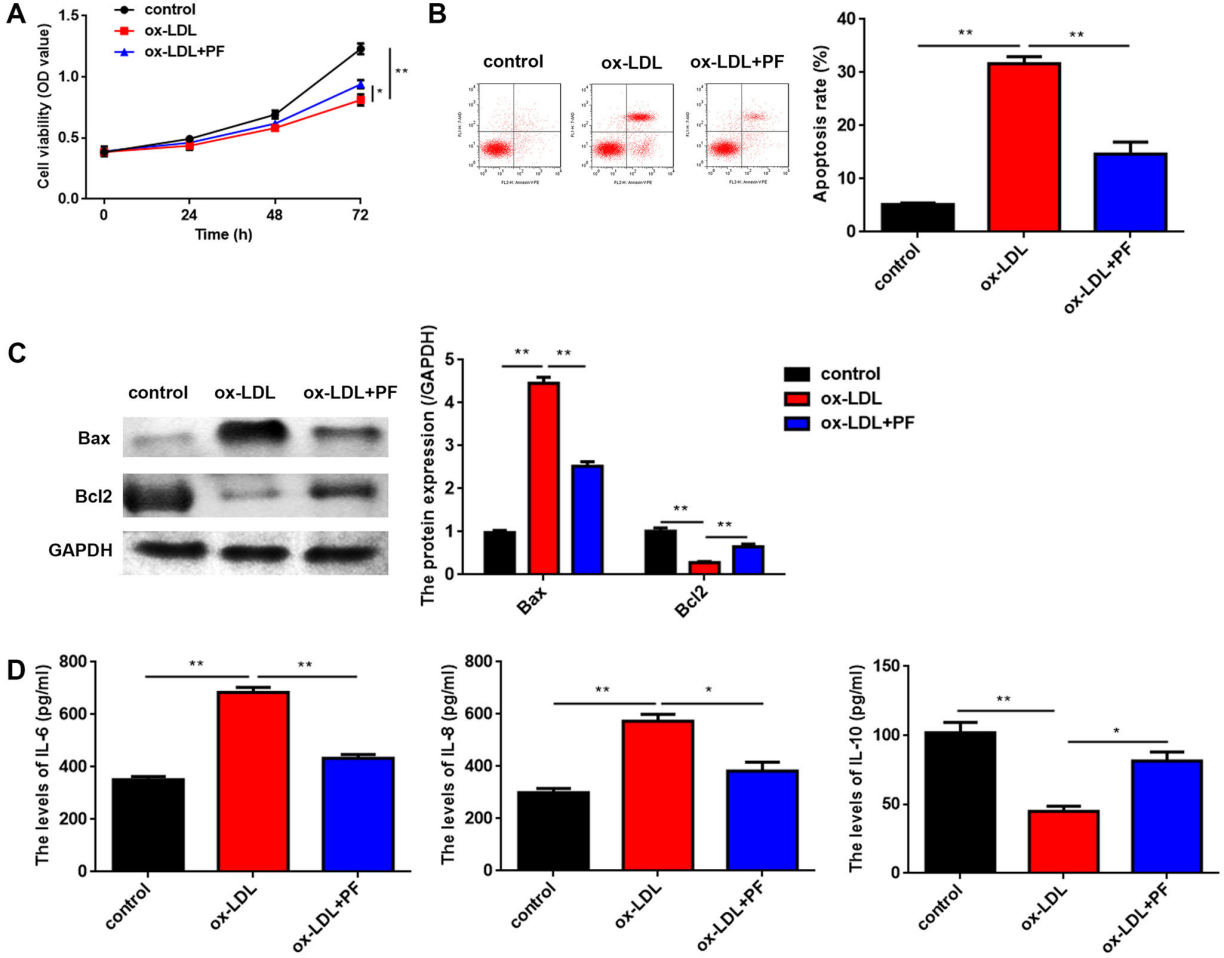
PF promotes cell viability and inhibits apoptosis and inflammation induced by ox-LDL. (A) Cell viability was analysed using the MTT assay; (B) cell apoptosis was analysed using flow cytometry; (C) the Bax and Bcl2 levels were examined using western blot and (D) the levels of IL-6, IL-8 and IL-10 were measured by ELISA. *p < 0.05 and **p < 0.01.

As shown in Figure 2(A), ox-LDL significantly inhibited cell viability, while PF reversed the inhibition. Meanwhile, PF suppressed cell apoptosis of ox-LDL-induced HCAECs (Figure 2(B)). Furthermore, ox-LDL significantly increased Bax levels and decreased Bcl2 levels, while PF markedly reversed the ox-LDL effects (Figure 2(C)). Moreover, the expression of inflammation-related factors IL-6 and IL-8 was upregulated, while that of IL-10 was reduced by ox-LDL, and PF treatment reversed the effects induced by ox-LDL (Figure 2(D)).

### PF suppresses the activation of the Wnt/β-catenin pathway

**Figure 3.**
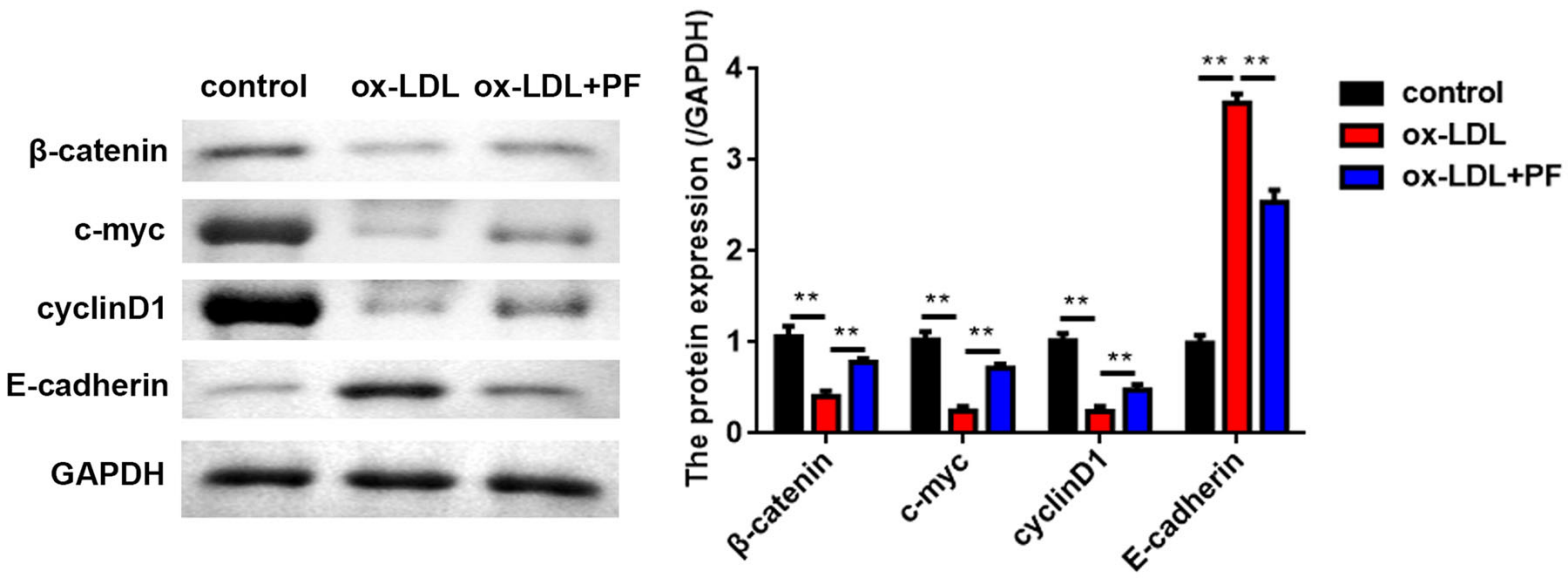
PF suppresses the activation of the Wnt/β-catenin pathway. The expression of Wnt/β-catenin pathway-related factors β-catenin, c-myc, cyclin D1 and E-cadherin was examined using western blot. GAPDH was the internal control. **p < 0.01.

The results of the western blot indicated that ox-LDL greatly reduced the levels of β-catenin, c-myc and cyclin D1 but markedly elevated the E-cadherin levels. However, PF abolished the effects induced by ox-LDL (Figure 3).

### PF suppresses cell apoptosis and inflammation by regulating the Wnt/β-catenin pathway

**Figure 4.**
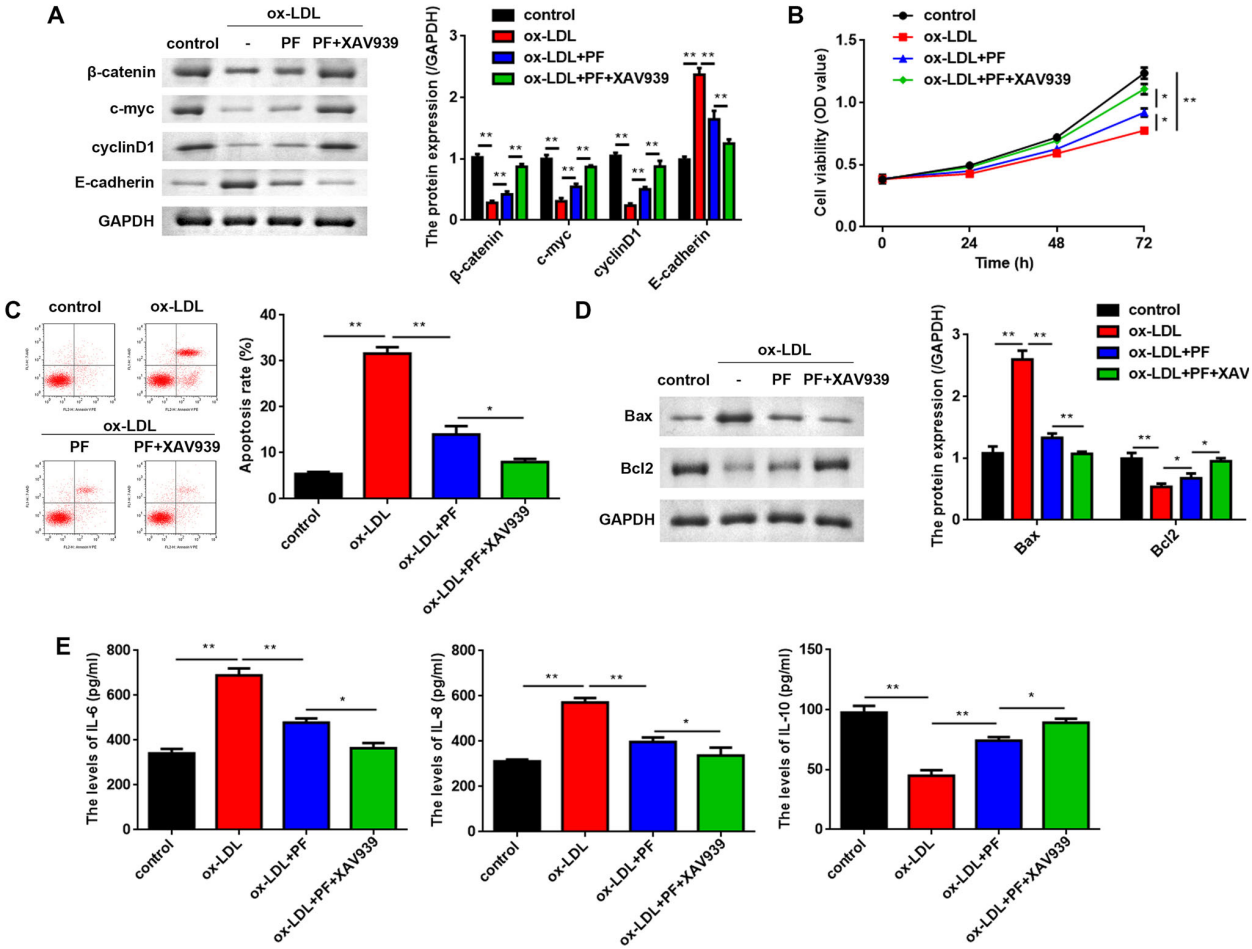
PF suppresses apoptosis and inflammation *via* regulating the Wnt/β-catenin pathway. (A) The protein levels of β-catenin, c-myc, cyclin D1 and E-cadherin were assessed using western blots; (B) MTT assay was conducted to assess cell viability; (C) flow cytometry was performed to analyse apoptosis; (D) the Bax and Bcl2 levels were examined using western blots, and (E) the levels of IL-6, IL-8 and IL-10 were determined by ELISA. *p < 0.05 and **p < 0.01.

To verify whether PF functions through the Wnt/β-catenin pathway, XAV939 (a Wnt/β-catenin pathway inhibitor) was used to treat the HCAECs, and it was found that XAV939 and PF co-treatment upregulated β-catenin, c-myc and cyclin D1 levels, but downregulated E-cadherin levels (Figure 4(A)). XAV939 further promoted the cell viability of PF-treated cells (Figure 4(B)). Conversely, PF reversed the promotion of apoptosis of ox-LDL-induced cells, and XAV939 further suppressed apoptosis (Figure 4(C)). The protein levels of Bax were notably decreased, whereas the Bcl2 levels were markedly increased in PF and XAV939 treated cells, compared with the ox-LDL + PF group (Figure 4(D)). Additionally, the levels of IL-6 and IL-8 were significantly reduced, while compared with the ox-LDL + PF group, the IL-10 levels were significantly elevated in the ox-LDL + PF + XAV939 group (Figure 4(E)).

## PF inhibits the formation of atherosclerosis in high-fat diet ApoE^−/−^ mice

**Figure 5.**
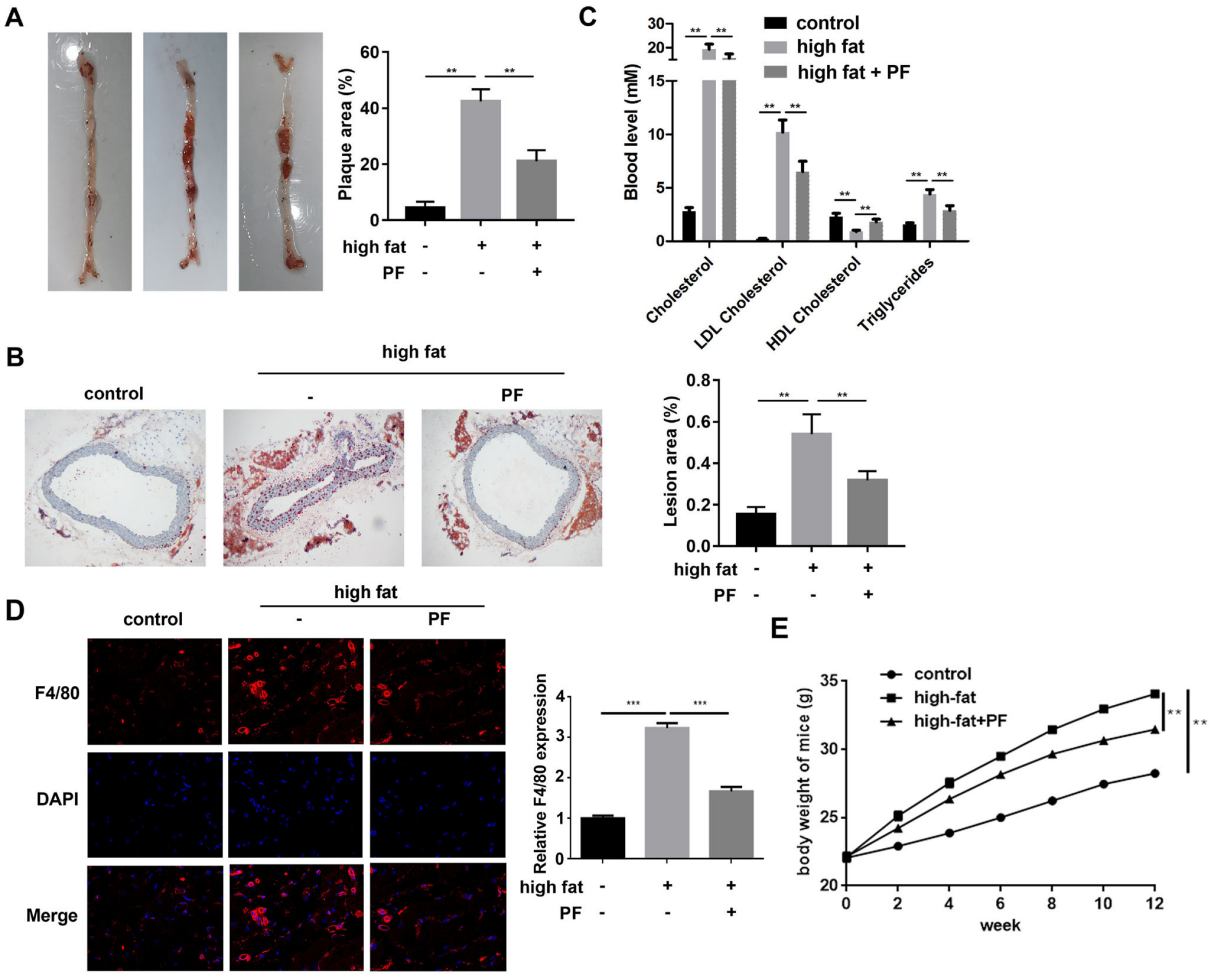
Effects of PF on the formation of atherosclerosis in ApoE^−/−^ mice. (A) The whole aorta was stained using the Oil red O, and the plaque areas (%) were quantified; (B) the lesion areas (%) of the aortic roots from mice of each group were examined using the Oil red O staining assay and quantified; and (C) the levels of cholesterol, triglycerides, LDL-cholesterol and HDL-cholesterol were detected using the specific kits. (D) The macrophage marker F4/80 was detected in aortic roots of mice using IF staining assay. (E) The body weight of mice was evaluated every two weeks. **p < 0.01 and ***p < 0.001.

The aorta and aorta root specimens of the ApoE^−/−^ mice were collected, and the pathogenesis was evaluated using the Oil red O staining assay. Compared with the control group, the mice in the high-fat diet group presented a larger plaque area, whereas PF reversed the effect and showed a smaller plaque area (Figure 5(A)). Additionally, the lesion area was larger in the mice of the high-fat diet group than that in the control group, and PF reduced the lesion area of the high-fat diet mice (Figure 5(B)). Besides, compared with the control group, the cholesterol, triglycerides, and LDL cholesterol levels were all increased, and the HDL cholesterol levels were decreased in the high-fat diet group, whereas PF reversed these effects induced by a high-fat diet (Figure 5(C)). Additionally, high-fat diet increased F4/80 expression in aorta root of mice, suggesting macrophage infiltration, whereas PF inhibited macrophage infiltration in high-fat diet mice (Figure 5(D)). As shown in Figure 5(E), high-fat diet induced the gradual weight gain in mice compared to the normal diet, whereas PF inhibited the increased body weight of mice with high-fat diet.

## Discussion

In the current study, it was first found that PF promoted cell viability and suppressed apoptosis and inflammation of ox-LDL-induced HCAECs. Moreover, PF affected the biological functions by the Wnt/β-catenin pathway.

Ox-LDL is crucial in the initiation and development of cardiovascular diseases, including hypertension, CAD and cerebral infarction (Trpkovic et al. 2015), which binds to corresponding receptors to activate lipid peroxidation, thereby leading to endothelial dysfunction, manifested by endothelial cell apoptosis and oxidative stress (Tang Y et al. 2016; Hu et al. 2020). Thus, HCAECs were hereby treated with ox-LDL to establish the cell damage model. The results indicated that ox-LDL suppressed the viability and induced apoptosis of HCAECs, suggesting that ox-LDL induced endothelial injury, which is consistent with the previous studies mentioned above.

PF is a main active ingredient isolated from the whole plants of the Paeoniaceae family, especially from *Paeonia lactiflora* Pall. In CAD, PF matters considerably in the vasodilation, anti-hyperlipidaemia, antithrombosis and protection of myocardial ischaemia. For example, PF protects against myocardial ischaemia-reperfusion injury by suppressing oxidant stress and inhibits the inflammation of atherosclerosis by regulating the TLR4-mediated NF-κB pathway (Li et al. 2017; Wu et al. 2020). Xiongshao Capsule with PF component can attenuate the elderly CAD and reduce the postoperative recurrence rate (Shang et al. 2011). In addition, PF contributes to reducing ventricular remodelling, thus enhancing heart function and alleviating acute myocardial infarction (Chen H et al. 2018). However, the underlying mechanism of the action of PF in diseases is rather complex. To study the role of PF in CAD, much emphasis was hereby placed on the effects of PF on the biological functions of ECs, including cell viability, apoptosis and inflammatory response, and it was found that PF facilitated cell viability and suppressed apoptosis of HCAECs induced by ox-LDL, suggesting the protective effect of PF on endothelial injury.

Nowadays, accumulating evidence has been reported that the occurrence and progression of CAD is identified as an inflammatory process of the arterial wall (Hansson and Hermansson 2011; Akyuz 2020). Inflammation also plays a crucial role in plaque rupture, which leads to angina pectoris and myocardial infarction (Ikonomidis et al. 2012). Additionally, multiple cytokines including pro- and anti-inflammatory factors are aberrantly expressed in endothelial cells during the progression of CAD (Tousoulis et al. 2015). IL-6 and IL-8 are important pro-inflammatory cytokines, whereas IL-10 is an anti-inflammatory cytokine. Herein, ox-LDL was found to induce the increase of IL-6 and IL-8 levels and the decrease of IL-10 levels in HCAECs. Moreover, PF treatment reversed the effects on IL-6, IL-8 and IL-10 expression induced by ox-LDL. All these findings suggested anti-inflammatory properties of PF during the progression of CAD.

Alteration of the Wnt/β-catenin pathway is closely related to numerous human diseases, such as cancers, CAD, type-2 diabetes and metabolic diseases (Weerackoon et al. 2021). In CAD, the activation of the Wnt signalling is associated with endothelial dysfunction, as well as biological functions of smooth muscle cells (Liu et al. 2020). For example, the activation of β-catenin facilitates apoptosis and thus inhibits neointima formation (Williams et al. 2016). The knockdown of H19 regulates the biological behaviours in ox-LDL-stimulated vascular smooth muscle cells by modulating the Wnt/β-catenin pathway (Zhang et al. 2018). Additionally, the Wnt/β-catenin pathway, provided with both anti-inflammatory and proinflammatory effects, is involved in regulating inflammatory and immune escape (Ma and Hottiger 2016). However, most evidence suggested that the inactivation of the Wnt/β-catenin pathway downregulated the levels of proinflammatory factors including IL-6, IL-8, TNF-α and IL-1β and upregulated anti-inflammatory factors such as IL-10 (Jang et al. 2019; Tan et al. 2021). Therefore, based on the importance of the Wnt/β-catenin pathway, we explored whether PF affects biological functions of HCAECs through mediating this pathway. The results indicated that the Wnt/β-catenin pathway was hereby activated by ox-LDL, which was inhibited by PF. Moreover, inhibiting the Wnt/β-catenin pathway further enhanced the protective effects of PF on the damaged HCAECs. These findings suggested that PF regulated the biological behaviours of HCAECs by inactivating the Wnt/β-catenin pathway.

Atherosclerosis is an important cause of CAD, characterized by abnormal lipid metabolism. Thus, the effects of PF on plaque lesions and serum lipid in the mice model were hereby studied. High-fat diet treated ApoE^−/−^ mice model was considered the ideal model to construct hypercholesterolaemia (Chen L et al. 2019) that induced increased plaque area of whole aorta, serum lipid markers and lesion area of aorta root in the present study. High triglyceride and LDL cholesterol levels and low HDL cholesterol levels were important markers of dyslipidaemia. HDL cholesterol was negatively related to CAD, and triglyceride was a risk factor of CAD (Fruchart and Duriez 2002). Inhibiting the transport of HDL cholesterol promoted the deposition of triglyceride and LDL cholesterol on the vascular wall (Taskinen 2003). Moreover, it was found that PF attenuated aortic lesions and macrophage infiltration, decreased cholesterol, LDL cholesterol, and triglycerides and increased HDL cholesterol *in vivo*, suggesting the efficiency of PT in decelerating the progression of CAD.

## Conclusions

The study first reveals that PF attenuates ox-LDL-induced apoptosis and inflammation by regulating the Wnt/β-catenin pathway and suggests the potential of PF for CAD therapy.

## Data Availability

The datasets used and/or analysed during the current study are available from the corresponding author on reasonable request.
